# Melanoma Single-Cell Biology in Experimental and Clinical Settings

**DOI:** 10.3390/jcm10030506

**Published:** 2021-02-01

**Authors:** Hans Binder, Maria Schmidt, Henry Loeffler-Wirth, Lena Suenke Mortensen, Manfred Kunz

**Affiliations:** 1 Interdisciplinary Center for Bioinformatics, University of Leipzig, 04107 Leipzig, Germany; binder@izbi.uni-leipzig.de (H.B.); schmidt@izbi.uni-leipzig.de (M.S.); wirth@izbi.uni-leipzig.de (H.L.-W.); mortensen@izbi.uni-leipzig.de (L.S.M.); 2 Department of Dermatology, Venereology and Allergology, University of Leipzig Medical Center, Philipp-Rosenthal-Str. 23-25, 04103 Leipzig, Germany

**Keywords:** melanoma, single-cell transcriptome sequencing, treatment response, pseudotime analysis

## Abstract

Cellular heterogeneity is regarded as a major factor for treatment response and resistance in a variety of malignant tumors, including malignant melanoma. More recent developments of single-cell sequencing technology provided deeper insights into this phenomenon. Single-cell data were used to identify prognostic subtypes of melanoma tumors, with a special emphasis on immune cells and fibroblasts in the tumor microenvironment. Moreover, treatment resistance to checkpoint inhibitor therapy has been shown to be associated with a set of differentially expressed immune cell signatures unraveling new targetable intracellular signaling pathways. Characterization of T cell states under checkpoint inhibitor treatment showed that exhausted CD8^+^ T cell types in melanoma lesions still have a high proliferative index. Other studies identified treatment resistance mechanisms to targeted treatment against the mutated BRAF serine/threonine protein kinase including repression of the melanoma differentiation gene microphthalmia-associated transcription factor (MITF) and induction of AXL receptor tyrosine kinase. Interestingly, treatment resistance mechanisms not only included selection processes of pre-existing subclones but also transition between different states of gene expression. Taken together, single-cell technology has provided deeper insights into melanoma biology and has put forward our understanding of the role of tumor heterogeneity and transcriptional plasticity, which may impact on innovative clinical trial designs and experimental approaches.

## 1. Melanoma Biology, Clinics and Treatment

Melanoma is a highly aggressive cutaneous neoplasia, which has been intensively analyzed by molecular techniques in the past few years [1,2]. Indeed, a number of mutational analyses have been performed and identified key driver mutations among which mutant *BRAF*V600 is the most prevalent, affecting almost 50% of all melanoma patients [3,4,5]. Mutant *BRAF*V600 leads to an activation of the classical mitogen-activated protein kinase (MAPK) pathway with downstream targets being mitogen-activated extracellular signal-regulated kinases (MEK) 1/2 and extracellular-signal regulated kinases (ERK) 1/2. Promising treatment responses were obtained by targeting this pathway on the level of BRAF kinase and MEK1/2, which has been a mainstay of melanoma therapy in recent years [1,2,6]. BRAF-targeted treatment includes small molecule inhibitors vemurafenib, dabrafenib, and encorafenib, directed against activated (mutated) BRAF kinase, which has significantly improved the survival rate of affected patients. Overall treatment success is hampered by the fact that a significant number of patients show primary resistance (~20%) and also secondary resistance, which occurs in the vast majority of patients. The currently used treatment options mainly consist of combination treatments of BRAF inhibition combined with MEK1/2 inhibition, which is not only more effective but also reduces side effects such as the development of epidermal neoplasias and exanthemas [7]. Cobimetinib, trametinib, and binimetinib are currently used as MEK1/2 inhibitors in combination therapy. The 5-year overall survival rate of combination therapy has reached 50%, which may be regarded as a major breakthrough for this highly aggressive tumor [8]. Still, the majority of patients develop a secondary resistance [9]. More recently, adjuvant treatment after complete tumor eradication in stage III (lymph node metastasis) has been approved using this combination of treatment, and neo-adjuvant (pre-operation) treatment studies are under way [10,11].

The mechanisms underlying primary and secondary resistance to targeted treatment have been an area of intensive investigations in recent years [12,13,14,15,16,17,18,19,20,21,22,23]. Among the most prominent cellular mechanisms are switches to *NRAS* mutations [15,17,23], aberrant *BRAF* splicing [14,15], *BRAF* amplifications [12,13,15,16,17], *MAP2K1* (*MEK1*) mutations [13,17], *PTEN* and *PIK3CA* mutations [17], and *COT* overexpression [21].

Principally, mechanisms of treatment resistance are heterogeneous but show many overlapping patterns in different studies. An earlier study found NRAS and PDGFR overexpression in a number of melanoma cell lines after development of treatment resistance to BRAF inhibitor PLX4032 in vitro [23]. In one of the most comprehensive subsequent studies, 45 patients were analyzed by whole-exome sequencing before BRAF inhibitor (vemurafenib or dabrafenib) treatment, after early (less than 12 weeks) and late development of resistance [17]. Top resistance variants were *NRAS* mutations, *BRAF* amplifications, *MEK1* and *MEK2* mutations, and *PTEN* mutations/amplifications. *MEK1* and *PTEN* mutations were partly already present before treatment.

A similar study analyzed 59 metastatic melanoma lesions from patients treated with dabrafenib or vemurafenib [15]. Authors performed a targeted genetic screen of *NRAS*, *BRAF*, *MEK1*, *MEK2,* and *AKT1* genetic variants. Resistance mechanisms were found in 58% of progressing tumors, with *BRAF* splice variants, *BRAF* amplifications, and *NRAS* and *MEK1/2* mutations being present in 8–32% of cases. In another study, *BRAF* amplifications were found in 4 out of 20 melanoma patients, and *NRAS* mutations in 5 out of 20 melanoma patients, under treatment with vemurafenib, as determined by whole-exome sequencing and quantitative polymerase chain reaction (PCR) [16]. *BRAF* genetic amplifications were also a major mechanism of secondary treatment resistance in a study on 28 melanoma samples resistant to combined BRAF and MEK inhibition, as determined by targeted sequencing of *BRAF*, *NRAS*, *KRAS*, *MEK1,* and *MEK2* [12]. Overall, 8 out of 28 samples showed very high numbers of *BRAF* amplifications (ultra-amplifications). Subsequent in vitro studies showed that *BRAF* ultra-amplified melanoma cell lines may become addicted to BRAF/MEK1 inhibition, as cells died after drug-removal [12]. This might be of relevance for clinical settings and might speak for drug holidays to enhance later treatment response.

In an analysis of 10 patients under combined treatment with dabrafenib and trametinib, genetic resistance mechanisms were found in 9 out of 11 progressing tumors analyzed by a focused PCR panel for *BRAF*, *NRAS*, *MEK1/2,* and *AKT1* [13]. *BRAF* amplifications were found in 4, and *MEK1/2* mutations in 3 samples. In a smaller study analyzing 5 patients that developed acquired resistance to combined BRAF and MEK1/2 inhibition (dabrafenib, trametinib), a new mutation in *MEK2* was observed in one patient, and *BRAF* amplification and alternative *BRAF* splicing were found in two other patients [14]. The pathogenic role of the new *MEK2* mutation was verified via in vitro experiments. *BRAF*-mutant melanomas may also acquire resistance through activation of the EGFR pathway, as shown in a study on melanoma patients treated with vemurafenib or dabrafenib, a finding that has been observed earlier in colon cancer [19]. *EGFR* expression was shown to be related to *SOX10* downregulation, and in vitro *SOX10*-low cells were enriched in the presence of BRAF inhibition in vitro. This phenomenon was reversed after drug removal, which also suggests drug-holidays being useful in re-sensitizing cells to BRAF inhibition. Taken together, the mentioned studies underline that resistance mechanisms in melanoma to targeted treatment use different mechanisms but center around re-activated MAPK or PTEN-AKT1 pathways.

Studies on transcriptional mechanisms of primary resistance to targeted treatment revealed a MITF-low/NF-κB-high transcriptional phenotype which could be linked to specific gene expression profiles in cell lines and patient biopsies, and a MITF-low/AXL-high phenotype [20,22]. In vitro, combined treatment of cell lines with BRAF inhibition and an AXL inhibitor significantly reduced melanoma cell viability of MITF-low/AXL-high cells, supporting the functional relevance of these findings [20]. In a large-scale study using RNA-sequencing of metastatic melanoma samples, transcriptomic patterns of 48 single-drug or double-drug disease progressors were compared with patient-matched baseline melanoma tissues [18]. Transcriptomic patterns of treatment resistance involved differential gene expression of tumor and stromal genes. Among up-regulated genes in resistant lesions were *c-MET*, *IL-8*, *c-FOS*, macrophage marker *CD163*, chemokine *CCL8*, and *NFKBIA*.

However, these transcriptional mechanisms are incompletely understood as the underlying data mostly originates from bulk sequencing studies; thus, they do not reflect clonal structures and are only partly recapitulating selection processes. Overall, primary and secondary resistance mechanisms to targeted therapy may be either due to genetic changes (mutations, amplifications) or changes in gene expression of specific pathways [24].

Major progress was made in the field of immunotherapies in melanoma. Immunotherapies, in particular immunecheckpoint inhibition (ICI), targeting the cytotoxic T lymphocyte antigen 4 (CTLA-4), programmed cell death protein 1 (PD-1), and programmed cell death protein ligand 1 (PD-L1) have been approved in recent years in a number of different cancers including malignant melanoma [25]. However, a larger number of patients did not respond to these treatments (60% in case of PD-1 inhibition, 80% for CTLA-4 inhibition) as a primary resistance. Treatment response rates could slightly be enhanced by a combination of anti-PD1 and anti-CTLA4 treatment [26].

The underlying mechanisms for primary and secondary treatment resistance to immunotherapies have been studied in recent years and include a number of different mechanisms [27]. In one of the earliest studies addressing this issue, immune markers of anti-CTLA4 treatment response of melanoma patients were analyzed [28]. Pre-treatment tumors of overall 110 patients were analyzed by whole-exome sequencing. Transcriptome data were generated for 40 patients. Mutational load, neoantigen load, and transcriptomes of cytolytic activity were associated with treatment response. Enhanced expression of granzyme A (*GRZMA*) and perforin 1 (*PRF1*) were associated with responses, as were *CTLA-4* and *PD-L2* expression. In a parallel study of another group, melanoma exomes from 64 patients treated with CTLA-4-blocking antibodies were analyzed by whole-exome sequencing [29]. Mutational load alone was not sufficient to predict treatment benefit. Neoepitope analysis identified neoantigen landscapes with a strong treatment response. Further, the predicted neoantigens were able to activate T cells from patients treated with anti-CTLA4 antibodies in in vitro experiments.

Hugo and co-workers performed a large-scale study on 28 metastatic melanoma lesions, 27 of which were pre-treatment lesions, and analyzed gene expression patterns of responding versus non-responding lesions [30]. Overall, the mutational load of tumors correlated with patient survival (but not with tumor response). Among the genes that were upregulated in non-responding lesions were mesenchymal transition genes such as *AXL*, *WNT5A*, and *TWIST*, as well as immunosuppressive genes such as *IL10*, *VEGFA,* and *VEGFC*. Resistant lesions showed a gene expression signature called IPRES (innate anti-PD1 resistance), which is comprised of 26 transcriptomic signatures including mesenchymal transition, wound healing, and angiogenesis.

In a first study on secondary treatment resistance, samples from paired baseline and relapsing lesions in four patients were analyzed [31]. By use of whole-exome sequencing it was shown that resistant lesions in two patients carried mutations in interferon-receptor-associated Janus kinase 1 (*JAK1*) or Janus kinase 2 (*JAK2*) genes, associated with the deletion of the wild-type allele. A third patient showed a truncating mutation in the beta-2-microglobulin (*B2M*) gene, which is part of the MHC-I complex.

In a later large-scale analysis of treatment resistance, 54 samples with CTLA4- blockade followed by anti-PD1 treatment were analyzed by a 12-marker immunohistochemistry panel and NanoString^®^ technology (of 795 immune-related genes) (nanoString, Seattle, WA, USA) [32]. This study showed that individual protein markers and gene expression patterns in early on-treatment biopsies were predictive of responses for the checkpoint blockage. Among markers for treatment response to anti-PD1 treatment were CD8, CD4, CD3, PD-1, PD-L1, and LAG3 protein expression in responders versus non-responders in early on-treatment samples. In the NanoString^®^ analyses of response to anti-PD1 treatment, up-regulation of HLA-molecules, IFN-γ pathway effectors, and different chemokines were observed.

In a subsequent study, 68 patients with advanced melanoma were investigated before and after anti-PD-1 treatment by whole-exome and transcriptome analysis [33]. Responders under treatment experienced a so-called mutation contraction, which means that the number of clonal and subclonal variants decreased on therapy in these patients. Transcriptomic analyses showed an increase in gene expressions patterns of CD8^+^ T cells, NK cells, and M1 macrophages in responders as compared to non-responders.

Recently, pre-treatment tumors taken from 144 metastatic melanoma patient were analyzed by whole-exome and whole-transcriptome sequencing, and mutational and transcriptomic features were assessed for correlation with response to anti-PD1 treatment [34]. Interestingly, there was no significant association of specific gene mutations to response or resistance to treatment. Regarding gene expression, 4 of the 13 MHC-II associated HLA genes were significantly upregulated in responders. Significantly enriched pathways in responders were IFN-γ response, allograft rejection, complement, inflammatory response, and interleukin (IL6)-JAK-STAT3 signaling. Signatures for T cells, B cells, macrophages, CD8^+^ cytotoxic, and exhausted CD4^+^ T cells were also enriched. Interestingly, there were differences between patients with previous exposure to anti-CTLA4 treatment and those who were naïve to this treatment with a higher expression of immune-related pathways in in responders of the anti-CTLA4 pre-treated group. Among prominent immune genes were *CXCL9*, *CXCL10,* and *CXCR3*, among prominent immune markers were CD20, CD163, CD4, FOXP3, and CTLA-4.

In a more recent study, authors built an immune predictive score called IMPRES, based on a gene pattern analysis of spontaneously regressing neuroblastomas, to predict immune checkpoint inhibitor response in melanoma [35]. Overall, 18 immune checkpoint genes were chosen and high expression of *HVEM* (a member of the tumor necrosis factor receptor superfamily), *CD27* and *CD40* were associated with better response rates, while immune inhibitory molecules such as *CD276*, *TIM-3*, and *VISTA* were associated with worse response. This signature was tested in an own melanoma data set of 41 patients of the authors under immune checkpoint blockage and on other data sets [30,32]. In particular, *PD-1*/*OX40L* expression was predictive for anti-PD1 treatment response. However, the predictive capacity of the IMPRES score is controversially discussed [36].

Taken together, a significant number of genetic and genomic studies on mechanisms of treatment response and resistance under immune checkpoint inhibition support the notion of immune cell patterns, HLA molecules and chemokines as major drivers of response.

Recurrences and treatment failures of melanoma may not only derive from global changes in gene patterns but also from intra-tumor heterogeneity and the outgrowth of pre-existing treatment-resistant clones. Moreover, evidence has also been provided that tumor cell plasticity mediated by an activated tissue microenvironment secreting TNF-α and other immune modulators may cause resistance [37,38]. Transcriptional and epigenetic states may change during treatment and impact on recurrences and treatment resistance [37,39].

Multiple subclonal mutations, gene expression patterns, or epigenetic mechanisms may be present in tumor lesions and create a genetically heterogeneous population of tumor cells. In addition, the tumor microenvironment can impact on melanoma biology, in particular on predisposed subclones or subclones that may be re-programmed transcriptionally. Here, we summarize current knowledge on the analysis of melanoma heterogeneity through single-cell RNA-seq (scRNA-seq) technology, with an emphasis on treatment response and resistance.

## 2. Clonal Heterogeneity in Melanoma

Clonal heterogeneity is currently regarded as one of the most relevant factors for treatment resistance and recurrence of malignant tumors [40]. The model of a clonal evolution of tumors with many molecularly heterogeneous subclones had been suggested in earlier reports, at a time when the molecular basis of tumor heterogeneity could not be analyzed in more detail [41]. Based on current knowledge, many of the recurrences and treatment failures of metastatic tumors derive at least in part from this clonal heterogeneity in tumor lesions consisting of different molecular subclones [42,43].

In melanoma, intra-tumor heterogeneity has been described for the presence or absence of *BRAF* mutations, but more detailed analyses on mutational patterns were still lacking at that time [44].

The molecular heterogeneity in melanoma lesions has been analyzed recently in more detail by use of whole-genome sequencing of distinct macrodissected tumor areas of primary melanomas and metastases [45]. In this study, 8 melanoma samples of primary melanomas and lymph node metastases were analyzed with multiregion sequencing of 41 regions. On average, 489 non-synonymous mutations were observed of which 13% were heterogeneously distributed. MAPK pathway genes (*BRAF*, *NRAS*, and *NF1*) were frequently mutated throughout all tumor regions (truncal mutations). Mutational tumor heterogeneity was associated with patient survival, with higher heterogeneity leading to shorter overall survival in this limited cohort of patients. Phylogenetic trees showed that 88% of driver mutations as derived from the catalogue of somatic mutations in cancer (Cosmic) database were truncal mutations, supporting their role in melanoma biology. Further analyses with larger sample sets and consecutive biopsies may help to understand the biological and clinical impact of this intra-tumor heterogeneity. Heterogeneously distributed mutations were found in subclones for *PIK3CA*, *PIK3R1*, *PTEN*, *MSN*, *JAK2*, *JAK3*, *NOTCH2,* and *IDH1*, with little overlap between the different samples.

In a subsequent study, authors compared mutational patterns of melanomas with signs of chronic sun damage (CSD melanomas) with high and low sun damage [46]. Ultra-deep sequencing was performed for 72 in situ and invasive melanomas for 40 cancer-associated genes. One sample set of an individual patient was analyzed in more detail regarding 5 regions in the primary tumor and 7 in in-transit metastases. There were no significant differences regarding the transcriptomes as determined by RNA-seq. Whole-exome sequencing showed that the vast majority of all mutation (96%) were found in all lesions and were regarded as truncal mutations, which comprised *KIT* and *CTNN1* mutations. In total, 60 genes were carrying non-truncal mutations, only four (*COL3A1*, *CTNNB1*, *FOXO3*, and *SRC1*) belonged to the Cancer Gene Census (https://cancer.sanger.ac.uk/census), and the majority was thus regarded as passenger mutations. Interestingly, phylogenetic trees showed that mutations in primary lesions did not appear earlier than in in-transit metastases.

In a recent paper on melanoma, tumor heterogeneity has been simulated in vivo by an admixture of 0.05% of A375 BRAF inhibitor-resistant melanoma cells to 99.95% of A375 BRAF inhibitor-sensitive melanoma cells [47]. This mixture was subcutaneously injected into mice. After treatment with the BRAF inhibitor vemurafenib, the number of resistant cells significantly increased in the overall regressing tumors, basically laying the foundations for relapse and secondary resistance of these tumors [47]. This phenomenon is in agreement with clinical findings for metastatic melanomas. However, based on current knowledge, selection of subclonal populations during treatment response are not the only mechanisms that support treatment resistance. Resistance may also derive from drug-induced re-programming [48].

The prognostic relevance of intra-tumor heterogeneity has recently been emphasized, as higher heterogeneity was associated with worse outcomes [49]. Authors used a clonal heterogeneity analysis tool (CHAT) to estimate intra-tumor heterogeneity, and CIBERSORT to analyze the immune cell composition from a cohort of 402 melanoma patients of TCGA [49,50]. More heterogeneous tumors were associated with gene patterns indicating less CD8^+^ T cells, T follicular cells, and M1 macrophages, while gene patterns of tumor-promoting M2 macrophages were enhanced. Highly heterogeneous tumors also had lower *PD1* and *PD-L1* expression and a lower expression of genes of cytotoxic pathways. These data were confirmed by others, showing that indeed intra-tumor heterogeneity plays an important role in patient survival, a finding that was further validated in a murine melanoma model with B16 melanoma cells [51].

Taken together, the mentioned studies have provided strong evidence for a prognostically relevant genetic heterogeneity in melanoma, either in primary lesions or in metastases. A deeper understanding of this heterogeneity may be achieved by recently developed single-cell technologies.

## 3. Single-Cell Technology

### 3.1. Principles of Single-Cell Individualization

Single-cell technology is based on the individualization of cells using different technologies. For a further in depth understanding, the reader is referred to a number of excellent recent reviews [52,53]. One of the first technologies addressing single-cell transcriptomics was introduced by the company Fluidigm^®^ (Fluidigm, South San Francisco, CA, USA). This technology is based on a complex microfluidics system. Single cells are captured on an integrated fluidic circuit RNA-seq chip within the Fluidigm^®^ C1 system. Cell capture, cell lysis, mRNA reverse transcription, and cDNA amplification are performed within the system. Subsequent next-generation sequencing may be performed by Illumina^®^ (Illumina, San Diego, CA, USA) sequencing technology [54,55]. This technology allowed the analysis of 96, and later 384, cells at a time. One major advantage of this technology lies in the fact that whole transcriptomes could be analyzed with complete mRNAs (in the 96-cell format). Other microfluidic systems were introduced, e.g., by 10xGenomics^®^ (10x Genomics, Pleasanton, CA, USA), based on a direct integration of single cells in a emulsion that harbors an individual cell, gel beads covered with molecular identifiers, and capture oligonucleotides. In principle, this technology allows an unlimited number of cells to be analyzed (5000–10,000 cells are recommended). Sequencing is limited to 3′-primed ends of the respective mRNAs and sequencing depth is generally lower compared to the Fluidigm^®^ system. Apart from these widely used techniques, a number of individually established technologies are in use in different laboratories. Recent technologies also include sampling of cells into multi-well plates with subsequent lysis and library preparation in a multi-well format using the SmartSeq 2^®^ (Illumina, San Diego, CA, USA) protocol to overcome 3′-end bias.

### 3.2. Single-Cell Data Processing and Analysis

**Figure 1:**
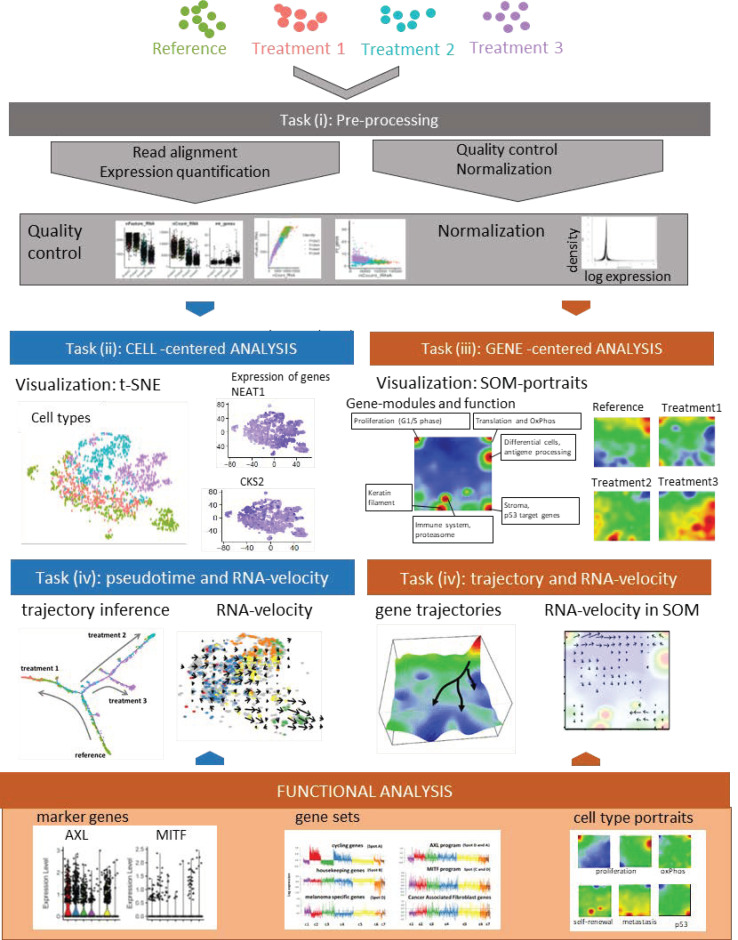
Principles of single-cell analyses based on melanoma cells analyses: A schematic representation of single-cell analyses is shown. Analysis starts with classical read alignment and quality control and normalization measures as in bulk RNA-seq. Subsequent analysis of transcriptomic cell clusters is performed by t-distributed stochastic neighbor embedding (t-SNE) as described in the text. Individual genes (*NEAT1*, *CKS2*) may be mapped onto clonal structures. Alternatively, self-organizing maps may be chosen, which represents a gene-based clustering method of cellular subclones. Pseudotime dynamics shows different trajectories of treatment resistance under three different treatment conditions which may also be demonstrated by RNA-velocity analysis. Marker genes for treatment resistance are found in different subclones and are part of larger gene sets or cell type portraits.

Bioinformatics analysis is crucial for extracting knowledge from scRNA-seq data in order to discover the heterogeneity of cell populations in space and time and to understand the underlying molecular mechanisms on tissue, cell and gene levels (Figure 1). A variety of tools have been designed to conduct bulk RNA-seq data analyses, but many of them cannot be directly applied to scRNA-seq [56,57]. Except short-read mapping, almost all data analyses such as differential expression, cell clustering, and gene regulatory network inference have certain disparities between scRNA-seq and bulk RNA-seq techniques. Because of the low amount of starting material, scRNA-seq has limitations regarding data quality due to comparatively low capture efficiency and high dropouts. It produces noisier and sparser data compared to bulk RNA-seq raising substantial challenges for computational analysis. Downstream analysis can be split into two orthogonal views, namely focusing either onto the cells as the functional unit or onto genes or gene programs. Whereas cell-based analysis commonly performs clustering and describes pseudo-temporal behavior of the data, gene-based analysis typically extracts differentially expressed genes, gene sets, and gene regulatory networks. In both situations, scRNA-seq also raises new conceptual challenges, namely to merge ‘traditional’ differential gene expression analysis of bulk samples with cell-type differentiation, diversity analysis, and counting, as performed traditionally using methods such as FACS (fluorescence-activated cell sorting) or using whole-genome gene expression as a marker instead of single fluorescent labels to identify a cell type. Such joint analyses of cell- and gene-level information provide novel insights regarding genomic regulation of cell-type specific programs and regarding cell-types, particularly in terms of continuously varying states during development of tissues in health and/or disease. Such views enable extracting (pseudo-)temporal information from cross sectional data, estimating interactions between different cell types on gene and molecular levels, and, in consequence, specifying tissue architecture, e.g., in terms of tumor microenvironment and physiological state of the bystander cells.

Bioinformatics analysis workflow of scRNA-seq data may be divided into five major tasks (i–v), which still raise a series of methodical and conceptual challenges and therefore is under permanent development [58].

i. Preprocessing aims at removing the effect of all factors without relevance for the expected biological effects. It includes read alignment, expression quantification, quality control, technical bias correction, and normalization. Mapping tools originally developed for bulk RNA-seq are mostly applicable also to scRNA-seq data. Further steps include quality control and filtering of unwanted genes and cells (e.g., cells expressing only a few genes); imputing missing values, batch correction (reducing systematic measurement biases between different runs and/or treatment groups), and normalizing gene expression (reducing unwanted variance between cells due to capture efficiency, sequencing depth, dropouts, and other technical effects). Technical noise of scRNA-seq is a common problem due to the low starting material and challenging experimental protocols. Detailed descriptions and recommendations of suited program-tools have recently been published.
ii. Cell typing and diversity analysis aims at disentangling cell identities and their functional impact in the respective tissues. It includes clustering of cellular transcriptomes and their assignment to cell types (also called populations) in a supervised or unsupervised way. The former classification approach uses cell-type gene signatures taken from previous studies to assign the new data to these ‘known’ cell types. The latter class-discovery approach splits new data into de-novo groups of cells. In a second step, these new groups are related to known cell types by applying previous cell type signatures and statistical enrichment techniques, thus linking unsupervised with supervised approaches. Classifying cells into types or physiological states is essential for many secondary analyses to characterize the tumor microenvironment by composition of immune cells and/or to extract varying fractions of tumor cells from different developmental stages. For this task and scRNA-seq in general, reliable reference systems with a resolution down to cell states are required. Depending on the research question, even intermediate transition states might be of interest. Reference cell atlases of cell types of different healthy and cancer tissues, of immune cells and of melanoma-related cell types extracted from previous melanoma studies, have been published in a number of reports [50,59,60] and have put an emphasis on immune cells in these settings [61,62,63].
iii. Gene module and marker extraction, functional analysis, and network inference aim at understanding gene regulation of cells on the gene level including aberrant effects due to genetic defects, external stimuli leading to treatment resistance and intrinsic evolutionary adaptations on tissue level. This task analyses co-expression of groups of genes, characterizes their functional context, and infers gene networks. Gene networks affect interactions between different cell types and/or signaling pathways. Beyond simple changes in average gene expression between cell types (or across bulk-collected libraries), scRNA-seq enables a high granularity of changes in expression. Particularly, cell type-specific alterations in cell state across samples are of special interest. These analyses deliver individual marker genes and sets of signature genes characterizing the different cell types and states, and, in addition, genes reflecting interactions between the cell types in the complex microenvironments of the respective tissue type. Appropriate methods to modularize transcriptional programs are non-negative matrix normalization (NMF) [64] or self-organizing-maps (SOM) [65].
iv. Analysis of developmental trajectories in terms of pseudotime and RNA velocity aim at deducing time-dependent aspects of tissue development and cancer progression from cross-sectional scRNA-seq data. scRNA-seq experiments provide snapshot data, which resolves the molecular heterogeneity of cell cultures and tissues with single cell resolution under static conditions (see task (ii)). Given, that each cell is measured only once, one needs computational methods to deduce developmental trajectories on cellular level from time-independent data. The pseudotime model assumes that single-cell transcriptomes can be understood as a series of microscopic states of cellular development that exist in parallel at the same (real) time in the cell culture or tissue under study. Moreover, the model assumes that the temporal development smoothly and continuously changes transcriptional states in small and densely distributed steps so that the similarity of transcriptional characteristics can serve as a proxy of time, called pseudotime. It scales development in units of values between zero and unities for the start and end points, respectively. The pseudotime algorithm typically aligns the cells along a trajectory in reduced multi-dimensional space where a large variety of projection algorithms can be applied, differing regarding criteria such as cellular ordering, topology, scalability and usability [66]. Each method has its own characteristics in terms of the underlying algorithm, produced outputs and regarding the topology of the pseudotime trajectory (e.g., predefined linear, multibranched, cyclic, or ‘inferred from the data’). ‘RNA-velocity’ provides another independent approach to infer developmental trajectories from static scRNA-seq data [67,68]. It directly ‘forecasts’ the transcriptional state of a cell based on the relation between spliced and unspliced mRNA in terms of a directional change of cell state in cell-diversity space (task (v)). RNA-velocity provides a vector-field reflecting transcriptional changes of each cell, which can be transferred into developmental trajectories joining sources and sinks of mRNA abundance in cell-state and gene-state space.
v. Dimension reduction, visualization of cell- and gene-state space, and data portrayals aim at enabling the intuitive perception of complex data in order to extract ‘hidden’ information and to support hypothesis development and testing. scRNA-seq data are high-dimensional data (ten-thousands of transcripts multiplied with ten-to-hundred thousand of cells multiplied with a multitude of biological conditions), which is difficult to visualize in its original form. Dimension reduction and appropriate visualization are therefore important challenges in all four tasks listed above. Conceptually, two perpendicular types of information have to be considered, namely cell- and gene-centered views on the scRNA transcriptomes as addressed in tasks II and III, respectively [61,69]. For the view on cell diversity, different methods projecting multidimensional cell-transcriptomes data into two dimensions are in use, such as Principal Component Analysis (PCA), t-distributed stochastic neighbor embedding (t-SNE) and Uniform Manifold Approximation and Projection (UMAP) [70,71]. These methods produce point clouds in cell similarity space visualizing mutual similarities between the single-cell transcriptomes in terms of colored clusters of cells (task II) and/or of colored expression levels of selected gene markers and signatures in the individual cell transcriptomes (task III). For visualization of transcriptomic landscapes in gene state space, we developed the expression portrayal method based on self-organizing map (SOM) machine learning. Such landscapes support identification of modules of co-expressed genes, of their mutual network topology and of their functional context [72,73].

### 3.3. Single-Cell Exome-Seq

Single-cell technology has also been applied to exomes. An earlier exome sequencing study on renal cell carcinoma identified 260 mutations in 25 single cells, but could not reveal a clonal structure in this tumor [74]. This may be due to the relatively small number of cells analyzed. Interestingly, many of the newly identified mutated genes had not been described before in renal cell carcinoma. In a whole-genome single-cell sequencing study on lymphoblastic leukemia, targeted sequencing was performed of a panel of single nucleotide variants (SNVs), deletions, and IgH sequences in 1,479 single tumor cells from six acute lymphoblastic leukemia (ALL) patients [75]. A clonal structure of the individual samples could be identified, consisting of 1 to 5 different clones.

A sequencing study for combined genomic DNA and mRNA analysis of the same cell was performed for a mouse embryonic stem cell line [76]. The discrimination between the DNA and mRNA libraries was relying on the use of two different amplification protocols and different molecular adaptors. Authors compared copy numbers with gene expression data and found that copy number variations appeared to influence variability in gene expression among cells. This study showed for the first time that parallel DNA and RNA analyses may in principle be possible for one individual cell, but it is still far from routine use. Overall, single-cell whole-genome or exome sequencing (exome-seq) is principally more challenging than scRNA-seq analysis due to the high number of amplification-derived artifacts. Some recent publications have described the key issues of this technology [77]. By use of single-cell exome-seq technology, a phylogenetic tree of metastasis development has been built for colon carcinoma [78]. Authors showed that metastasis development appeared to be a late event in primary tumors after accumulation of a larger set of mutations. Interestingly, there was no obvious overlap regarding the accumulation of mutations before metastasis in different samples, and the first metastasis-specific mutations also did not overlap between samples. Authors used a FACS-based technology to separate their cells. A more recent study used exome-sequencing technology for the analysis of a limited number of genes using the Tapestri^®^ (Mission Bio, South San Francisco, CA, USA) platform to unravel the clonal diversity of T-cell acute lymphoblastic leukemia (T-ALL) [79]. The panel contained 110 genes and tested more than 100,000 cells of 25 samples. Longitudinal samples showed clones with a minor presence at diagnosis that, at later stages, developed into relevant major clones. This technology may be of particular relevance for hematological malignancies but may also be used in solid tumors.

## 4. Single-Cell Analyses in Melanoma

### 4.1. Primary Melanomas, Lymph Node Metastases and Cell Lines

**Figure 2:**
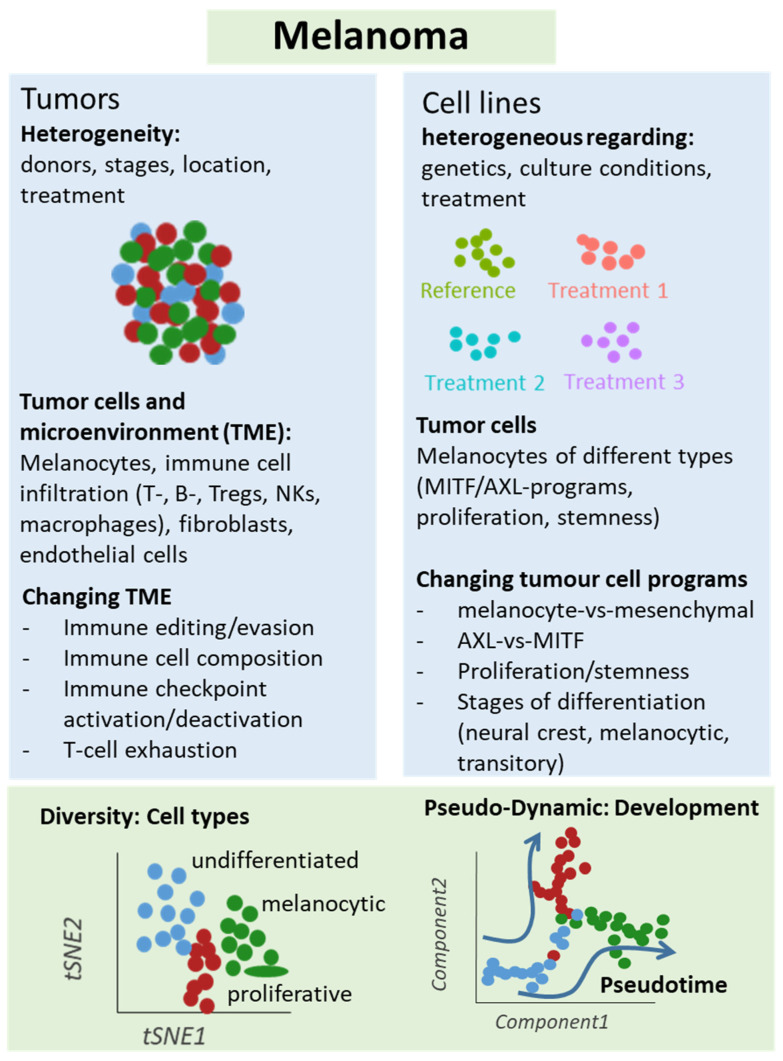
Schematic representation of single-cell analyses in melanoma samples. Either melanoma tumors or cell lines underwent single-cell sequencing analyses, which unraveled different types of cells and levels of heterogeneity in melanoma lesions and mechanisms of treatment resistance, e.g., mediated by T cell exclusion or T cell exhaustion programs under immune checkpoint therapy. Changes in cellular programs in tumor cells were also identified in cell culture studies supporting a role of the dichotomy of the AXL and MITF programs for treatment response and resistance.

scRNA-seq has been applied in a significant number of studies on malignant melanoma (Table 1; Figure 2). The first major scRNA-seq study analyzing melanoma tissues investigated 19 samples of primary melanomas and metastatic lesions [80]. A significant inter-tumor heterogeneity was observed for melanoma cells in these tissues, while the immune cells in these analyses showed a relative homogeneous gene expression pattern [80]. Major subgroups of transcriptional heterogeneity were associated with cell cycle, spatial context of cells, and a drug-resistance program (MITF-low/AXL-high signature). Authors used single-cell signatures of T cell, B cells, fibroblasts, macrophages, and endothelial cells derived from this study and mapped them onto gene patterns of bulk sequences of 471 melanoma samples present in the Cancer Genome Atlas (TCGA). They then searched for genes expressed by cells of one type that may influence or reflect the proportion of cells of a different cell type in the tumor. By this means they showed that the abundance of cancer-associated fibroblasts (CAF) is predictive of the phenotype, and that fibroblast signatures influence the presence of specific immune cell signatures. Moreover, they identified single cells with an AXL-high/MITF-low signature in an AXL-low/MITF-high population, which would have been missed in bulk sequencing and may give rise to treatment resistance. The AXL/MITF dichotomy has been supported by a later study re-analyzing these data by a new software called Cyclum to identify latent periodic developmental processes [81].

Our group has analyzed the transcriptomes of 92 single cells cultured from a patient biopsy of a *BRAF* wild type/*NRAS* wild type melanoma metastasis by scRNA-seq [65]. We used self-organizing maps (SOM) to identify sub-clones and found gene patterns of proliferation, oxidative phosphorylation, pigmentation, and cellular stroma [65]. These categories could be further refined, especially regarding cell cycle genes referring to different stages of cell cycle such as G1, S, and G2/M phase. In principle, every cell showed an individual gene pattern. Interestingly, gene expression patterns overlapped with those of clinical gene expression studies with associated patient survival data, further emphasizing the clinical relevance of these single-cell analyses [82]. Cellular heterogeneity was less pronounced in *BRAF* mutant/*NRAS* wild type and *BRAF* wild type/*NRAS* mutant cultures. In order to identify new treatment options based on gene expression patterns, kinome expression patterns across sub-populations were analyzed. Cell cycle kinases CDK4 and CDK2 were consistently highly expressed in a majority of cells, suggesting that both might be interesting targets. Indeed, treatment of melanoma cell cultures cells with CDK4 inhibitor palbociclib reduced cell proliferation to a similar extent as MAPK inhibitors. Finally, a low abundant subclone with high expression of an ABC transporter module, surface markers CD271 and CD133, and multiple aldehyde dehydrogenases (ALDHs), was identified. These findings support a role of cancer stem cells in melanoma biology, which has been described for other tumor entities [90,91]. Thus, single-cell gene expression patterns may provide new treatment targets above BRAF/MAPK inhibitor treatment.

In another report, authors addressed the question whether melanoma cells may shift between different states of differentiation from a melanocytic on one end and a mesenchymal-like state at the other end of differentiation [82]. Authors studied 10 melanoma cultures using scRNA-seq and found shared gene regulatory networks that underlie the extreme melanocytic and mesenchymal states which are both present in an intermediate state. Among both states SOX9 (de-differentiated stage) and SOX10 (differentiated stages) play an important role. The intermediate state shared some transcriptional regulons such as SOX10, TFAP2, and MITF with the melanocytic state, but also with the mesenchymal state such as FOSL1, IRF3, and STAT1. The transcriptional state had functional consequences, as it determined the migratory capacity of cells. After *SOX10* knockdown, cells monitored in time-series and analyzed by pseudotime analysis, showed a sequential transition between both extreme states (with SOX10 and SOX9 expression). Thus, this intermediate state indeed exists and is characterized by a distinct gene regulator network rather than by heterogeneous mixture of cells, further supporting the notion of a transcriptional plasticity between different melanoma cell states.

### 4.2. Treatment Resistance Under Immune Checkpoint Inhibition

Analysis of treatment resistance and response has been a focus area of a number of single-cell studies [83,84,85,88]. In one of the earlier studies, scRNA-seq analyses were performed for 33 human melanoma tumors, and a T cell signature was identified that allowed to classify hundreds of bulk-sequenced tumors as tumors harboring a seed (T-cell)-exclusion program [84]. This exclusion program was then mapped onto ipilimumab and anti-PD1 treated samples analyzed by scRNA-seq to identify co-expressed genes in individual cells. The T cell exclusion program included transcriptional patterns of apoptosis, JAK-STAT3 signaling, TNF pathway, senescence-association, Myc targets, and p53 binding. Indeed, melanoma lesions expressed features of this program as determined by multicolor immunofluorescence. Among the genes tested were *TP53* (up), *JUN* (down), *MYC* (up), and *HLA-A* (down). The presence of the treatment resistance program also correlated with checkpoint inhibitor response in melanoma patients in an independent sample set. Finally, blockage of CDK4/CDK6 kinases, which were part of the resistance program, inhibited melanoma cells growth in vitro and in vivo in a melanoma mouse model. Taken together, single-cell analyses allowed the identification of a malignant cell program that is associated with T cell exclusion and is predictive for checkpoint inhibitor resistance that maybe targeted by CDK4/CDK6 inhibition.

In another study, single-cell transcriptomes were generated via 48 tumor samples from 32 metastatic melanoma patients using a Smart-seq2 protocol [83]. Biopsies were taken at baseline and under anti-PD1 inhibitor treatment, either alone or in combination with anti-CTLA4 treatment, including two patients with anti-CTLA4 treatment alone. CD45^+^ cells were used to define an 11-cluster transcriptomic pattern. Two clusters were more prominent in responder lesions and in non-responder lesions. Treatment resistant clusters were enriched for genes linked T cell exhaustion (*LAG3*, *PDCD1*, *HAVCR2*, *TIGIT*, *CD38*) and cell cycle genes (*CDK1*, *CCNB1*, *MKI67*, and *CDK4*). Individual markers of responders were *LTB*, *TCF7*, and *CCR7*, and of non-responders were *CCL3*, *CD38*, and *HAVCR2*. Authors then focused their analyses on CD8^+^ T cells, which consisted of two clonal states, memory/survival state, and exhaustion state, called CD8_G and CD8_B cells, respectively. Prominent populations of response were TCF7^+^/CD8^+^ T cells (TCF7 is part of Wnt signaling and crucial for differentiation and self-renewal). Further analysis via cell-sorting showed that CD39/TIM3 discriminated exhausted from memory/effector cells, which was verified in a B16-F10 murine melanoma model. T cell receptor (TCR) analysis showed that enriched TCRs were more common in exhausted clusters. Together, this study may help to select patients for anti-PD1 therapy based on subclonal T cell states, which may be of relevance for clinical trials.

A more recent study focused on dysfunctional T cells in melanoma lesions under immune checkpoint therapy [85]. In this study, 25 melanoma samples were analyzed by scRNA-seq with a focus on CD4^+^ and CD8 T^+^ cells in patients with prior treatment against CTLA-4 or PD-1, or a combination of both. Immune cell subtypes were widely shared across patients, but their relative abundance differed considerably between patients, even when disease stage and treatment background were matched. In particular, CD8^+^ T cells partly transitioned into a dysfunctional T cell pool characterized by the expression of *PDCD1*, *LAG3*, and molecules shared with CD4^+^ Treg (e.g., *CSF1*, *ZBED2*). Interestingly, however, single-cell TCR sequencing and expression of cell cycle genes showed that these so-called dysfunctional T cells had the highest levels of clonal expansion. Ex vivo cultured tumor-infiltrating lymphocytes (TIL) from melanoma patients showed that tumor reactivity correlated with a CD8^+^ T cell dysfunctional state. Collectively, these data suggests that the dysfunctional CD8^+^ T cells are dynamically differentiating and are an active cell compartment with tumor reactivity in patients. Models of regulation of this T cell compartment should help to create innovative treatment approaches.

These data were partly re-analyzed (8 non-treated melanoma patients) in a subsequent study [92]. Here, authors addressed the question of a spatiotemporal activity of IFN-γ as a major mediator of tumor immunity. First, a Myc-driven B cell lymphoma and a B16F10 murine tumor model were used. When injecting OVA antigen-positive and negative B16F10 melanoma cells into mice, mosaic tumors were generated. By co-injection of CD8^+^ OVA-specific T cells, a homogenous expression of MHC class I, and PD-L1 upregulation on tumor cells was induced, irrespective of a close proximity of T cells and melanoma cells, supporting the notion of a distant activity of immune cells. As mentioned, by reanalysis of the abovementioned clinical single-cell data, it was shown that CD8^+^ T cells are indeed the major source of IFN-γ, and interferon-signatures were found in different cell populations such as macrophages and neutrophils (melanoma cells were sparse in these samples). These findings suggest that tumor cells in human melanomas might also be targeted by distant immune cells in the microenvironment.

### 4.3. Treatment Resistance under Targeted Treatment

To identify markers of resistance against BRAF inhibitor (BRAFi) treatment, a new analysis software was developed that outperformed existing platforms regarding large complex substructures and large numbers of sampled cells [64]. The software was termed SAKE, which stands for scRNA-seq analysis and klustering evaluation. To further test this software, melanoma cell lines 451Lu and A375 were analyzed either as parental or BRAF inhibitor (vemurafenib)-resistant cells. t-SNE analysis showed that four populations may be separated, but individual cell clones with gene patterns of parental cells existed in resistant cells and vice versa (without obvious enrichment). By use of differential gene expression analysis the major differentially expressed gene with upregulation in resistant cells was shown to be *DCT* (dopachrome tautomerase). Sorting of cells from parental cells revealed that DCT-enriched cells were more resistant to BRAF inhibition than the whole culture. In a final set of experiments, authors observed a transitional population characterized by *ENT5A*, *AXL*, *GFR*, *PDGFRB,* and *JUN* expression, which was localized at the tip of the parental population proximal to the resistant population, indicating a pre-resistant state as described earlier by others [48]. Without prior knowledge, SAKE identified this intermediate population, characterized by *AXL*, *JUN*, *NGFR*, *WNT5A*, *FGFR1*, and *NRG1* expression. By testing of copy number variations in single cells as a surrogate marker for genetic heterogeneity, it was found that this transitional (pre-resistant) population was derived from several unrelated clonal lineages and most likely reflects a transient stage rather than a particular clone. Taken together, *DCT* was identified as a marker for individual resistant clones in untreated populations and together with mechanisms of transient gene regulation towards resistance may be an interesting therapeutic target.

In another report, CRISPR RNA-guided deaminase technology was combined with CROP-seq (CRISPR droplet sequencing) technology to introduce mutations in 3 genes of the MAPK pathway in A375 melanoma cells, namely *NRAS*, *KRAS* and *MAP2K1* (*MEK1*) [87]. Overall, 420 sgRNAs were introduced into the melanoma cells. Subsequent drug response was tested against BRAF inhibitor vemurafenib. Enrichment of individual sgRNAs under treatment indicated treatment resistance. Most positive findings referred to sgRNAs targeting *MEK1* in the E203 codon region, which is a well-known resistance region. Apart from this, the most significant results were observed for mutations induced in the vicinity of Q61 in the *KRAS* gene, which has already been shown in other studies. Further transcriptomic analysis of resistant clones showed enhanced expression for *CD74*, *HLA-DRA*, *SLC26A2*, *HLA-DRB1*, *FOS,* and *HLA-DPA1* for *MEK1*E203K-related clones and *CXCL1*, *IL-8*, *CXCL2*, *SOD2*, and *CCL2* for *KRAS*Q61-related clones. Taken together, this study established a new platform that may be extended to other target genes and tumor entities to uncover mechanisms of resistance to targeted treatment.

In a recent report, an experimental setting is described recapitulating minimal residual disease after targeted treatment. In this study, subcutaneously injected mice with *BRAF*V600E mutant melanoma cells were treated with BRAF inhibitor dabrafenib [86]. Single-cell analyses were performed for minimal residual disease after transplanted tumors regressed under treatment. In the subclonal structure of minimal residual disease, for 4 different cell states were enriched such as NCSC (neural crest stem cells), invasive cells, SMC (starved-like melanoma cells), and pigmented cells, among which SMC showed the most significant enrichment under treatment. Single cell trajectories as derived from pseudotime analyses showed that an early proliferative state developed via two different developmental trajectories into NCSC and SMC cells. By use of multiplexed immunohistochemistry (IHC) of melanoma lesions, it was demonstrated that murine melanoma xenografts showed a specific spatial pattern for the different cell states marked by different discriminative markers. In further in vitro experiments, the transition into the NCSC state was cell-autonomous and reversible, tested by drug exposure and subsequent drug removal. Finally, computational analysis of gene regulatory networks showed that another component of the gene regulatory network of minimal residual disease is the retinoid X receptor-γ. Consequently, inhibition of this receptor by the small molecule inhibitor HX531 led to significantly longer survival of mice under dabrafenib treatment, with a significant percentage (20%) of mice being tumor-free even after 4 months of treatment. Together, treatment resistance appears to develop along different developmental trajectories identified by single-cell transcriptomics, a finding which may be exploited for new treatment approaches.

In a study on BRAFi treatment of different melanoma cell lines, RNA-seq data of 18 melanoma cell lines were included [88]. Nine cell lines were used to define different levels of BRAFi sensitivity. Drug resistant cells showed a low melanocytic cell signature, and elevated levels of neural crest and mesenchymal genes as well as genes of activated JNK and NF-κB pathways. Based on the analysis of different *NGFR* and *MRT-1* expression, a cluster of highly plastic cell lines under BRAFi treatment of brief (3d) or prolonged (71–90 d) inhibition was defined. These cells developed signatures of NCSC and epithelial-to-mesenchymal transition genes and genes of elevated invasiveness and migration. Using a Markov model for prediction, authors showed that the clusters underwent both cell state interconversion and drug selection. In a subsequent single-cell analysis, single-cell barcode chip technology (SNBC) was used to analyze 13 different proteomic markers (including NGFR, TNFR, MART-1, JNK, pERK, and pNF-κB p65). It was demonstrated for one of these cell lines that BRAFi treatment increased cellular heterogeneity at day 3 and 6 of treatment, which was reminiscent of cell state transitions in other systems [93]. There was a negative correlation between NGFR and MITF/MART-1 expression at day 3. At day 6, an activation of MEK/ERK and NF-κB p65 signaling was observed, suggestive for a role of both pathways for an adaptive cell state transition. Indeed, by use of specific inhibitors (trametinib, MEK inhibitor; and JSH23, NF-κB p65 translocation inhibitor), an additional growth arrest was found in these cells under BRAFi treatment. However, a combination of vemurafenib and trametinib did not halt the neural crest transition, and resistance emerged after prolonged treatment. Only the triple combination of vemurafenib, trametinib, and the JSH23 inhibitor kept the cells in a drug-sensitive state, which argues for the strong role of NF-κB p65 in treatment resistance.

These experiments were further extended in a more recent study of the same group, analyzing a larger number of parameters in single cells by use of the same microchamber technology [89]. Among these parameters were MITF, pERK1, p-NF-κB, KI67, NGFR, HIF1α, LDH, and glucose. A BRAF-mutant melanoma cell line was treated with BRAFi, and analyses were made at different time points (days/D0, D1, D3, and D5). Trajectories of BRAFi resistance were measured. Two different trajectories (upper and lower) were observed, characterized by either Ki67 and NGFR/AXL expression or MITF, MART-1 expression. Authors then isolated MITF-high and MITF-low cells from this cell culture and treated cells with BRAFi. Both cell types again used different trajectories for treatment resistance. Together, these results suggest that, upon drug treatment, MITF-high and low cells use distinct trajectories of treatment resistance. Finally, critical point analysis was performed. Here, “critical point” stands for a point of irreversible development. Two different cell clusters were identified that characterized the regions near such tipping points of both trajectories. One cluster showed high network connectivity in a pathway that included MITF, PFK, p-LKB, PKM-2, and LDH-2, while in the other cluster, TNFR, N-cadherin, and p-NF-κB were dominant. Consequently, inhibition of PKM2 and NF-κB with specific inhibitors showed different sensitivities in both cell types, and a combination of both inhibitors with BRAFi was more effective than double combinations. Taken together, the different heterogeneous drug-response trajectories improved our understanding of resistance development, which may have an impact on effective therapy combinations in future.

## 5. Spatial Sequencing in Melanoma

In order to disentangle the spatiotemporal organization of single-cell analyses, recent experimental approaches tried to analyze near-single-cell transcriptomes in situ. By use of so-called spatial transcriptomics, gene expression patterns were generated from tissue biopsies and analyzed by classical next-generation sequencing [94,95]. Indeed, spatial resolution does not allow single-cell cell resolution, but is at present limited to an area of 100 × 100 micrometers per spot. Further technological advances will surely go into the direction of single-cell resolution. For melanoma, a study about four lymph node metastases, based on an individual array system, has been published [94]. Overall, 286 tissue domains were analyzed per section (as duplicates), and a total of 2200 domains were investigated that measured 3000 transcripts per domain. Two different gene panels (factors) were observed in the four samples. Melanoma-A consisted of *CD63*, *PMEL*, and *S1000A1*, while Melanoma-B consisted of *S100B*, *FTH1*, and *AEBP1* expression. The factors were heterogeneously distributed among the 4 samples. Melanoma A was present only in samples 1, 2, and 4. Mapping of the gene expression patterns of 284 spots revealed 4 functional clusters, which overlapped with areas annotated by histopathology. Functional clusters included stromal tissue, tumor tissue, lymphoid tissue, and lymphoid tissue at the tumor border. Four genes were used to generate spatial heatmaps mapping to the 4 tissue clusters. This work showed that spatial sequencing in melanoma appears to be possible and provides reasonable results. Further refinement is needed to improve spatial resolution and sequencing depth.

In a more recent work, a more advanced spatial transcriptomic technology was applied to skin squamous cells carcinoma [96]. Transcriptomes of more than 8000 spots across 12 sections were analyzed, and 967 genes per spots were obtained. Spot expression patterns were consistent with the gross histologic architecture of lesions including tumor keratinocytes, tumor stroma, uninvolved stoma and adnexal areas, cancer-associated fibroblasts, and endothelial cells. The 10x Genomics^®^ Visium Spatial Gene Expression Kit^®^ was used for two additional patients with higher resolution. The leading edge of these tumors was composed of two different populations of tumor-specific keratinocytes and basal tumor cells. An immune landscape could further be defined, and it was shown that PD-L1 and PD-L2 were exclusively expressed by migrating dendritic cells (DC) in the tumor vicinity. These analyses identified multiple cell types involved in immunosuppressive mechanisms in DC, exhausted T cells, and Tregs, allowing for a refinement of the local tumor structures. In an extension of these analyses using the NicheNet software (https://github.com/saeyslab/nichenetr; GitHub, Inc., San Francisco, CA, USA)), molecules for specific cell-cell interactions were predicted [97]. Thus far, no such study has been published for primary melanoma lesions.

## 6. Single-Cell Sequencing of Copy Number Variations

Single-cell exome-sequencing has been performed in melanoma cell line COLO829 across 1475 cells [98]. Analysis was done using the chromium single-cell CNV solution (10× Genomics^®^) to sequence gDNA. Individual cells exhibited extensive copy number differences showing that this cell line was at least composed of 4 major clusters. Overall, 114 copy number variations were identified with ploidies of 2, 3, and 4, respectively. Chromosomal aberrations were observed for different chromosomes, e.g., for chromosome 18, present in group A and D, and a loss in groups B and C. Subclones emerged from chromosomal losses and gains. Taken together, in line with bulk sequencing reports, major chromosomal aberrations form in melanoma cells in a time-dependent manner, giving rise to heterogeneous cell populations.

## 7. Perspectives

The published data and further improvements of the mentioned technologies hold great promise for the analysis of melanoma and other tumors in the future. Moreover, the limited availability of fresh tumor tissue for many tumors will profit from the single-cell- analysis of frozen archival material with technologies that are currently under development [99]. Spatial sequencing will not only provide information about tumor heterogeneity but also unravel the spatial composition of different cell populations, which may be of particular relevance for immune checkpoint inhibitor treatment. Finally, emerging techniques of single-cell exome-sequencing, with the advent of customized technologies will further improve our knowledge about the emergence of resistant clones with specific genetic features, as has already been shown in hematological malignancies.

## Figures and Tables

**Table 1:** Summary of single-cell melanoma transcriptomics and proteomics studies and main outcome.

No	Melanoma Samples	Experimental or Clinical Set-Up	Characteristics of Clonal Structure	Main Findings	References
1.	Primary melanomas and metastases (*n* = 19)	Untreated	Clonal signatures of cell cycle, spatial context, drug-resistance programs	Presence of AXL-high/MITF-low population in a AXL-low/ MITF-high cluster; single-cell signatures with prognostic relevance	[80]
2.	Melanoma cell lines representing different stages of differentiation (*n* = 8)	Untreated	Cell clones with SOX9 and SOX10 high expression and transitional cells, knockdown of SOX10 affects clonal structure	Transition between gene networks instead of selection of individual clones (transcriptional plasticity)	[82]
3.	Melanoma short-term cultures (BRAF and/or NRAS mutant) (*n* = 3)	Untreated	Clonal structure of cell cycle, stromal, OxPhos, pigmentation genes	Four different clonal structures with additional subclonal structures and stem cell-like subclones	[65]
4.	Samples from 32 metastatic melanoma patients (*n* = 48)	Anti-PD1 inhibitor treatment of patients, either alone or in combination with anti-CTLA4 treatment	CD8^+^ T cells clones consisted of memory/survival (TCF7^+^) and exhaustion (CD38^+^) clones, respectively	TCF7^+^/CD8^+^ T cells are crucial for treatment response	[83]
5.	Human melanoma samples (*n* = 33)	Clinical samples under anti-CTLA4 treatment	Clonal immune exclusion program: CDK4/CDK6 expression, JAK-STAT3 signaling, TNF pathway, senescence-associated programs, Myc targets	CDK4/CDK6 inhibitor treatment of resistant clones improved survival of mice in a murine melanoma model	[84]
6.	Human melanoma samples (*n* = 25)	Anti-PD-1 inhibitor treatment of patients, either alone or in combination with anti-CTLA4 treatment	CD4^+^/CD8^+^ T cells with clusters of resting, transitional and exhausted T cells	Dysfunctional (exhausted) CD8+ T cells are still proliferative and showed tumor reactivity ex vivo	[85]
7.	Tumor tissue of melanoma cell line mouse xenografts (minimal residual disease) (*n* = 3)	Murine xenograft model, BRAFi treatment	Minimal residual disease with 4 different transcriptional subpopulations (pigmented, SMC, NCSC, invasive cells)	Enrichment of neuronal stem cells population after BRAFi treatment; successful treatment with retinoid receptor inhibitor	[86]
8.	A375 and 451Lu melanoma cell lines (*n* = 2)	BRAFi treatment	Patterns of resistance are present in parental cells and vice versa	Identification of a pre-resistant state at the tip of the parental population	[64]
9.	Melanoma cell line A375 (*n* = 1)	BRAFi treatment after CRISPR/Cas interference with MAPK pathway	Clonal selection of treatment resistant clones	Resistance-mediating positions in MAPK genes were mostly located around *MEK1*E203K or *KRAS*Q61	[87]
10.	BRAF-mutant melanoma cell lines (*n* = 3)	BRAFi treatment; testing of 13 different proteomic markers with single-cell barcode chip technology	Increased clonal heterogeneity under treatment	Activation of MEK/ERK and NF-κB p65 signaling in resitant clones; NF-κB inhibitor increased sensitivity of cells	[88]
11.	BRAF-mutant melanoma cell line (*n* = 1)	BRAFi treatment; testing of 19 different proteomic markers with single-cell barcode chip technology	Drug-induced clonal cell states changes with NGFR/AXL or MITF, MART1 patterns	Two different trajectories of treatment resistance of MITF-high and MITF- low cells	[89]

**Abbreviations:** BRAFi, BRAF inhibitor; SMC, starved-like melanoma cells, NCSC, neural crest stem cells; MAPK, mitogen-activated protein kinases; NGFR, nerve growth factor receptor; MITF, microphthalmia-associated transcription factor.

## Data Availability

Data available in publicly accessible repositories. For details, see cited references.
